# Antibody Positive Miller-Fisher Syndrome and Acute Motor Sensory Axonal Neuropathy With Respiratory Failure: A Rare Overlap

**DOI:** 10.7759/cureus.96422

**Published:** 2025-11-09

**Authors:** Rochan Athreya Krishnamurthy, Sukhvinder S Digpal, Ishaque Rafai, Drishya Kanhangad

**Affiliations:** 1 Internal Medicine, Portsmouth Hospitals University NHS Trust, Portsmouth, GBR; 2 Internal Medicine, Portsmouth Hospital University NHS Trust, Portsmouth, GBR; 3 Internal Medicine, University Hospitals Coventry and Warwickshire NHS Trust, Coventry, GBR

**Keywords:** acute motor and sensory axonal neuropathy (amsan), albumino cytogenic dissociation, anti-ganglioside antibodies, guillain-barre syndrome (gbs), invasive mechanical ventilation, miller-fisher syndrome (mfs)

## Abstract

Miller-Fisher syndrome (MFS), a rare variant of Guillain-Barré syndrome (GBS), is classically defined by ophthalmoplegia, ataxia, and areflexia and is strongly associated with anti-GQ1b antibodies. We report a 35-year-old man presenting with classical MFS features who rapidly deteriorated with progressive limb weakness and respiratory failure requiring mechanical ventilation. Cerebrospinal fluid analysis showed albuminocytologic dissociation, while nerve conduction studies were consistent with an axonal process. Serologic testing revealed positivity for both anti-GQ1b and anti-GD1a antibodies, confirming an overlap between MFS and acute motor sensory axonal neuropathy (AMSAN). The patient showed marked neurological recovery following intravenous immunoglobulin therapy and intensive supportive care. This case highlights a rare MFS-AMSAN overlap with respiratory failure and underscores the importance of early recognition, vigilant respiratory monitoring, and prompt immunotherapy in preventing morbidity and optimizing patient outcomes in rapidly progressive GBS-spectrum disorders.

## Introduction

Guillain-Barré syndrome (GBS) represents a heterogeneous group of acute, post-infectious, immune-mediated polyneuropathies that typically follow a preceding illness and progress rapidly to their clinical nadir, usually reflecting peak weakness or maximal symptom severity within four weeks. It has an estimated annual incidence of 1-2 cases per 100,000 population and encompasses variants such as acute inflammatory demyelinating polyradiculoneuropathy, acute motor axonal neuropathy, acute motor and sensory axonal neuropathy (AMSAN), and Miller-Fisher syndrome (MFS) [1].

MFS is classically defined by the triad of ophthalmoplegia, ataxia, and areflexia, and is strongly associated with anti-GQ1b IgG antibodies, which are detected in up to 85% of cases [2]. While MFS typically follows a benign and self-limiting course, it shares immunopathogenic overlap with other GBS variants, allowing for occasional transition or coexistence with more severe subtypes such as AMSAN. AMSAN, in contrast, is an aggressive axonal variant of GBS characterised by rapidly progressive motor and sensory deficits, areflexia, and a high likelihood of respiratory involvement. Clinical overlap between MFS and AMSAN, although rare, can manifest as a rapid neurological deterioration following an initially mild cranial neuropathy presentation. 

This case report describes a patient who initially presented with classical MFS features but rapidly progressed into an AMSAN overlap variant requiring mechanical ventilation. This case underscores the dynamic and rapidly progressive nature of GBS-spectrum disorders. It highlights the importance of early recognition, close respiratory monitoring and timely initiation of immunotherapy being vital for optimising outcomes.

## Case presentation

A 35-year-old man with no significant past medical history of note presented to the Emergency Department with complaints of progressive generalised malaise and weakness for three days. He had no smoking or alcohol consumption history and worked as a self-employed tattoo artist. 

During nurse-led triage, he was alert and oriented with a temperature of 36.6°C, blood pressure of 158/98 mmHg, heart rate 125 beats per minute, respiratory rate 20 breaths per minute, and oxygen saturation 98% on room air. His Glasgow Coma Scale (GCS) was 15/15. Over the next five hours, his condition deteriorated rapidly with worsening shortness of breath. He now required 15 litres per min of oxygen delivered via a non-rebreathe mask to maintain an oxygen saturation of 96%. His respiratory rate increased to 24 breaths per minute, heart rate to 132 beats per minute, and blood pressure to 170/94 mmHg, whilst his GCS remained unchanged. 

Further detailed history revealed a mild upper respiratory tract infection three weeks earlier and a brief, self-limiting diarrheal illness one week prior to his admission. He then developed a three-day history of progressive diplopia, distal paraesthesia, gait imbalance, dysphagia, dysarthria, mild hoarseness of voice, and progressive limb weakness. 

On examination, his airway was at risk due to copious secretions unremitting to suctioning. He demonstrated increased work of breathing and bilateral basal crackles on chest auscultation. Heart sounds were normal. A focused neurological assessment demonstrated normal muscle tone throughout, symmetrical weakness in both the upper and lower limbs, with strength graded as 4/5 on the Medical Research Council (MRC) scale. Deep tendon reflexes were absent in all four limbs. Plantar responses were flexor bilaterally. Cranial nerve involvement was evident with bilateral ptosis, mild bifacial weakness, and sluggish pupillary reactivity on the left side. Corneal and gag reflexes were absent. Sensory testing demonstrated grossly reduced fine touch sensation throughout the upper and lower limbs. Chest X-ray and ECG were normal. 

Due to progressive respiratory deterioration and airway compromise, he was intubated and ventilated immediately and transferred to the intensive care unit (ICU). Initial differential diagnoses included GBS and its variants, particularly MFS, due to the presence of classical ophthalmoplegia, ataxia, and areflexia. Further differentials included Bickerstaff brainstem encephalitis, brainstem stroke, intracranial lesion, and meningitis. Empirical antimicrobial therapy was initiated pending further investigations. 

Routine blood tests were grossly unremarkable except for a raised white cell count, while cerebrospinal fluid (CSF) analysis revealed features consistent with albuminocytologic dissociation (Table 1 and Table 2).

**Table 1:** Initial blood investigations and results.

Category	Test	Result	Reference Range
Full Blood Count (FBC)	Haemoglobin (Hb)	165 g/L	(130–170 g/L male, 120–155 g/L female)
	White Cell Count (WCC)	26.7 × 10⁹/L	(4–11 × 10⁹/L)
	Neutrophils	23.6 × 10⁹/L	(2.0–7.5 × 10⁹/L)
	Lymphocytes	1.4 × 10⁹/L	(1.0–4.0 × 10⁹/L)
	Platelets	423 × 10⁹/L	(150–400 × 10⁹/L)
Renal Function	Urea	9.2 mmol/L	(2.5–7.8 mmol/L)
	Creatinine	78 µmol/L	(60–110 µmol/L)
	eGFR	> 90 mL/min/1.73 m²	(> 90 mL/min/1.73 m²)
	Sodium (Na⁺)	136 mmol/L	(135–145 mmol/L)
	Potassium (K⁺)	4.9 mmol/L	(3.5–5.0 mmol/L)
Liver Function	Total Bilirubin	6 µmol/L	(≤ 21 µmol/L)
	ALT (Alanine aminotransferase)	32 U/L	(≤ 45 U/L)
	ALP (Alkaline phosphatase)	91 U/L	(30–130 U/L)
	Total Protein	72 g/L	(60–80 g/L)
	Albumin	25 g/L	(35–50 g/L)
Bone/Metabolic Profile	Corrected Calcium	2.29 mmol/L	(2.15–2.55 mmol/L)
	Glucose	6.5 mmol/L	(fasting 3.5–5.5 mmol/L)
Inflammatory Marker	C-reactive protein (CRP)	8 mg/L	(<5 mg/L)

**Table 2:** CSF results depicting albuminocytologic dissociation.

CSF Test	Result	Reference Range
Glucose	4.6 mmol/L	(typically ≥ 60% of plasma glucose)
Total Protein	0.61 g/L	(normal: 0.15–0.45 g/L)
Lactate	2.09 mmol/L	(1.1–2.4 mmol/L)
White Cell Count (WCC)	< 2 cells/μL	(0–5 cells/μL)
Viral PCR	Negative	Herpes simplex virus, Varicella zoster virus, and Enterovirus all negative

A non-contrast computerised tomography (CT) head showed no acute intracranial abnormalities. Serological testing for HIV, Hepatitis B and C, Epstein-Barr virus, Cytomegalovirus, syphilis, and Borrelia were also negative. Stool culture grew Campylobacter Jejuni species, and serological testing detected Mycoplasma Pneumoniae IgG.

Based on the characteristic clinical triad of ophthalmoplegia, ataxia, and areflexia, together with supportive CSF findings, a diagnosis of MFS was established in consultation with the neurology team. Subsequently, intravenous immunoglobulin (IVIG) was commenced at a total dose of 2 g/kg, administered over five days. Concurrent magnetic resonance imaging (MRI) of the brain was unremarkable, effectively excluding brainstem inflammation, stroke, or other intracranial pathology. A CT chest-abdomen-pelvis scan was done which excluded any deep source of infection and antibiotics were tailored to address a probable aspiration pneumonia.

A nerve conduction study (NCS) and needle electromyography (EMG) was performed 16 days after symptom onset and six days post IVIG therapy. Motor NCSs demonstrated reduced compound muscle action potential (CMAP) amplitudes with preserved distal latencies, conduction velocities, and F-wave latencies (Table 3), while sensory studies showed reduced sensory nerve action potential (SNAP) amplitudes with preserved peak latencies and conduction velocities (Table 4), consistent with an axonal process rather than demyelination. Needle EMG revealed no spontaneous activity with normal motor unit morphology and recruitment, signifying the absence of active denervation. These findings were in keeping with an AMSAN variant of GBS.

**Table 3:** Motor nerve conduction studies showing reduced compound muscle action potential (CMAP) amplitudes with preserved distal latencies, conduction velocities, and F-Wave latencies.

Nerve Tested	Site/Segment	Distal Latency (milliseconds)	Amplitude (CMAP, millivolts)	Conduction Velocity (meters per second)	F-Wave Latency (milliseconds)
Median (Right)	Wrist to Abductor Pollicis Brevis (APB)	3.4	7.3	—	28.4
Median (Right)	Elbow to Wrist	8.54	5.2	53.5	—
Tibial (Left)	Ankle to Abductor Hallucis (Abd Hal)	3.73	4.8	45.2	—
Ulnar (Right)	Wrist to Abductor Digiti Minimi (ADM)	2.44	4.6	—	25.9
Ulnar (Right)	Above Elbow to Wrist	7.82	3.3	53.9	—

**Table 4:** Sensory nerve conduction studies showing reduced sensory nerve action potential (SNAP) amplitudes with preserved peak latencies and conduction velocities.

Nerve Tested	Site/Segment	Peak Latency (milliseconds)	Amplitude (SNAP, microvolts)	Conduction Velocity (meters per second)
Median (Left)	Digit III to Wrist	2.13	0.59	56.3
Median (Right)	Digit II to Wrist	2.27	2.2	61.7
Superficial Peroneal (Left)	Calf to Medial Dorsal Cutaneous Nerve	2.15	4	37.2
Superficial Peroneal (Right)	Calf to Medial Dorsal Cutaneous Nerve	1.99	4.2	40.2
Radial (Right)	Web Space I-II to Wrist	1.52	13.4	55.9
Sural (Left)	Mid-Lower Leg to Lateral Malleolus	1.75	2.7	45.7
Sural (Right)	Mid-Lower Leg to Lateral Malleolus	2.33	4.7	42.9

Furthermore, serological testing for anti-ganglioside antibodies, performed at an external reference laboratory, subsequently returned positive for anti-GQ1b IgG and anti-GD1a IgG (both titres were elevated at >1000; laboratory reference: negative <500), confirming an overlap between MFS and AMSAN. 

He was successfully extubated after 13 days of mechanical ventilation and was able to mobilise with assistance two days later. A follow-up neurological examination after completion of IVIG therapy demonstrated mild improvement in symptoms. Muscle power increased to 4+/5 in the right upper and lower limbs and remained 4/5 in the left upper and lower limbs on the MRC scale. Deep tendon reflexes remained globally absent, and plantar responses were flexor bilaterally. Sensory examination showed persistent but improving reduction in light touch sensation, extending from the right hand to the wrist, from the left wrist to the mid-forearm, and from the feet to the knees bilaterally. Cranial nerve function showed gradual recovery, with improved diplopia and speech, although bilateral ptosis and a right facial droop were still evident. He had ongoing blood pressure fluctuations due to autonomic dysregulation thought to be secondary to the illness which later stabilised. The nasogastric tube inserted during the intensive care stay was removed following favourable speech and language therapy team assessments. He continued to report neuropathic pain affecting both the upper and lower limbs. He was transferred to a neuro-rehabilitation unit for intensive physiotherapy and eventually discharged home two months later. 

At one-month post discharge, the patient had near complete resolution of symptoms and had regained independence in almost all activities of daily living. Residual fatigue and subtle deficits in fine motor coordination of the hands persisted, limiting his occupational function as a tattoo artist. Nonetheless, he remained optimistic and demonstrated ongoing functional improvement. 

## Discussion

MFS is characterised by the classic triad of ophthalmoplegia, ataxia, and areflexia. It belongs to the “anti-GQ1b antibody spectrum” of GBS-related disorders, with anti-GQ1b IgG antibodies present in approximately 80-85% of cases. These antibodies are highly specific, and nerve conduction studies are often normal or show only mild reductions in SNAP or H-reflexes. Routine neuroimaging is usually normal [2]. 

In contrast, AMSAN is an axonal variant of GBS that presents with rapidly progressive, symmetrical weakness and sensory loss accompanied by areflexia. It is typically associated with anti-GD1a and anti-GM1 IgG antibodies. Electrophysiological studies demonstrate reduced CMAP and SNAP amplitudes with preserved conduction velocities, consistent with axonal degeneration [3]. 

Both MFS and AMSAN are primarily clinical diagnoses, supported by albuminocytological dissociation, antibody positivity, and characteristic nerve conduction findings [2,4]. In this case, the initial diagnosis of MFS was based on clinical evaluation, supported by CSF findings, prompting early initiation of treatment before additional results were available. The subsequent development of profound limb weakness and respiratory compromise, alongside electrophysiological evidence of axonal involvement with serologic positivity for anti-GD1a antibodies, established an overlap with AMSAN. 

In our patient, serum positivity for Mycoplasma pneumoniae IgG corresponded with a preceding upper respiratory tract infection approximately three weeks before symptom onset, while stool culture positivity for Campylobacter jejuni aligned with a brief self-limiting diarrhoeal illness one week prior. Together, these findings support an acute post-infectious, immune-mediated polyneuropathy consistent with GBS and its variants. Although Mycoplasma pneumoniae has been reported as a trigger for GBS and its variants, Campylobacter jejuni remains the most well-established antecedent infection, particularly in association with the axonal subtypes of GBS, and is also recognised as a common infectious trigger for MFS [2,5]. 

Autonomic dysfunction is a recognised feature of GBS and can lead to labile cardiovascular parameters, including episodes of tachycardia and blood pressure instability [5]. In this case, the initial rise in heart rate and blood pressure was attributed to autonomic involvement, likely compounded by sympathetic activation secondary to worsening respiratory distress. Consistent with this, our patient continued to experience fluctuating blood pressure throughout his ICU stay, which was closely monitored during rehabilitation and gradually stabilised prior to discharge.

This case highlights that the diagnosis of GBS variants, including MFS and AMSAN, should be guided primarily by clinical suspicion, as early CSF and nerve conduction studies may yield inconclusive results. A delay in timely management can be detrimental and should not be deferred while awaiting confirmatory results [5]. 

Isolated MFS generally follows a self-limiting course with an excellent prognosis. Persistent or life-threatening respiratory compromise is uncommon; contemporary literature reviews note complete resolution of ophthalmoplegia, ataxia, and areflexia by approximately six months on follow-up [2]. Nevertheless, rare cases can deteriorate to respiratory failure requiring ventilatory support [6]. By contrast, AMSAN, an aggressive axonal variant of GBS, has been shown in multicentre cohorts to carry a markedly higher risk of respiratory failure requiring mechanical ventilation, prolonged ICU and hospital stay with delayed functional recovery, reflecting its more severe clinical course compared to demyelinating or cranial variants [7]. 

This pattern was consistent with our patient’s progression, in whom initial MFS features evolved into an AMSAN, culminating in respiratory compromise and a more protracted recovery trajectory. This case underscores the potential for overlap between MFS and axonal GBS variants such as AMSAN, as well as the possibility of one evolving into the other. Both phenomena, although rare, have been described in the literature and highlight the importance of vigilant monitoring in patients with progressive or changing neurological manifestations [8,9]. 

The possibility of an evolving or unmasked axonal variant should be strongly considered in the differential diagnoses of any patient presenting with an MFS-like phenotype but later developing into a rapidly progressive weakness with respiratory compromise. This is particularly pertinent when an apparently isolated MFS presentation progresses over several days into a more severe GBS subtype [10]. Therefore, close inpatient observation of all patients with MFS is warranted until definitive signs of clinical stabilisation, with vigilant monitoring for any evidence of progression toward more aggressive variants precipitating respiratory compromise. 

The Erasmus GBS Respiratory Insufficiency Score (EGRIS) is a validated clinical prediction tool designed to estimate the risk of respiratory failure requiring mechanical ventilation in patients with GBS during the first week of hospitalisation. Developed and prospectively validated by Walgaard et al. in 2010, it has become a widely adopted instrument for early risk stratification and ICU planning in both research and clinical practice [11]. In our case, however, the patient’s rapid clinical deterioration left insufficient time to apply this scoring system, although its use remains valuable in guiding timely critical care decisions. 

In our case report, the combination of classic clinical features, rapid symptom progression, and early CSF findings prompted a timely suspicion of MFS with a possible overlapping GBS variant. This early clinical recognition allowed initiation of appropriate treatment without waiting for confirmatory anti-ganglioside antibody testing or nerve conduction studies. Prompt multidisciplinary team involvement, including neurology consultation, and rapid initiation of immunotherapy likely contributed to the patient’s swift neurological recovery and earlier weaning from mechanical ventilation than predicted [12]. 

## Conclusions

This case highlights the dynamic nature of GBS spectrum disorders, particularly the potential of MFS evolving into a more severe axonal GBS variant such as AMSAN, resulting in respiratory failure. Although MFS is relatively rare and generally benign, clinicians should maintain a high index of suspicion for overlap syndromes when patients demonstrate rapid neurological decline, especially rapid limb weakness and respiratory involvement, not in keeping with classic MFS features. This is more significant in acute care settings including the emergency department and acute medical units, as early diagnosis, vigilant respiratory monitoring, multidisciplinary team involvement and prompt initiation of IVIG can be lifesaving. 
